# Lidocaine inhibits staphylococcal enterotoxin-stimulated activation of peripheral blood mononuclear cells from patients with atopic dermatitis

**DOI:** 10.1007/s00403-013-1339-4

**Published:** 2013-04-16

**Authors:** Qingqing Jiao, Honglin Wang, Zhenglin Hu, Yin Zhuang, Weiqin Yang, Ming Li, Xia Yu, Jianying Liang, Yifeng Guo, Hui Zhang, Xilan Chen, Ruhong Cheng, Zhirong Yao

**Affiliations:** 1 Department of Dermatology, Xinhua Hospital, Shanghai Jiaotong University School of Medicine, 1665 Kongjiang Road, 200092 Shanghai, China; 2 Shanghai Institute of Immunology, Institute of Medical Sciences, Shanghai Jiao Tong University Medical School, Shanghai, China; 3 Department of Biochemical Pharmacy, School of Pharmacy, Second Military Medical University, Shanghai, China

**Keywords:** Atopic dermatitis, Staphylococcal enterotoxin A, Staphylococcal enterotoxin B, Lidocaine, Peripheral blood mononuclear cells, Activation

## Abstract

Atopic dermatitis (AD) is an inflammatory, chronically relapsing, pruritic skin disease and lesions associated with AD are frequently colonized with *Staphylococcus aureus (S. aureus)*. Activation of T cells by staphylococcal enterotoxins (SE) plays a crucial role in the pathogenesis of AD. Previous studies have demonstrated that lidocaine could attenuate allergen-induced T cell proliferation and cytokine production in peripheral blood mononuclear cells (PBMCs) from asthma patients. The purpose of this study was to investigate the effects of lidocaine on SE-stimulated activation of PBMCs from AD patients. PBMCs were isolated from ten AD patients and stimulated by staphylococcal enterotoxin A (SEA) or staphylococcal enterotoxin B (SEB) in the presence or absence of lidocaine in various concentrations. Cellular proliferation and the release of representative T_H_1- and T_H_2-type cytokines were measured. The effect of lidocaine on filaggrin (FLG) expression in HaCaT cells co-cultured with SE-activated PBMCs was also examined. Our results demonstrated that lidocaine dose-dependently inhibited the proliferative response and the release of IL-4, IL-5, IL-13, TNF-α, and IFN-γ from SEA- and SEB-stimulated PBMCs and also blocked the down-regulation of FLG expression in HaCaT cells co-cultured with SEA- and SEB-activated PBMCs. These results indicate that lidocaine inhibited SEA- and SEB-stimulated activation of PBMCs from patients with AD. Our findings encourage the use of lidocaine in the treatment of AD.

## Introduction

Skin lesions associated with atopic dermatitis (AD) are frequently colonized by *Staphylococcus aureus (S. aureus)* [23], which secrete two kinds of major staphylococcal enterotoxins A (SE), staphylococcal enterotoxin (SEA) and staphylococcal enterotoxin B (SEB), that are characteristic of staphylococcal superantigens (SsAgs) and bind to major histocompatibility complex class II molecules and selectively activate T cells expressing certain T cell receptor (TCR) Vβ-chain families. Therefore, SEA and SEB can precipitate or aggravate cutaneous inflammation in AD by induction of T cell proliferation and cytokine secretion [32, 43]. Reportedly, SEB-stimulated peripheral blood mononuclear cells (PBMCs) from AD patients and increased T_H_2 cytokine production were compared with healthy controls [19]. The T_H_2 cytokine milieu in AD is now thought to play a significant role in the destruction of skin barrier function [13]. In addition to superantigen activity, SsAgs also have been shown to induce inflammation and exacerbate disease activity by production of superantigen-specific IgE in patients with AD [37]. There have been various reports correlating disease activity in AD with superantigen production and with specific levels of anti-superantigen IgE [24, 26, 27]. Furthermore, SsAgs can inhibit the suppressive ability of regulatory T cells [3]. Thus, SsAg-induced T cell proliferation and subsequent cytokine production can be considered as a target in the management of AD.

AD is a chronic and relapsing T cell-mediated inflammatory skin disorder [8], which is likely driven by epidermal barrier dysfunction of the damaged skin and impaired host immune responses [42]. Filaggrin (FLG) is essential for the epidermal barrier formation and integrity and *FLG* gene mutations are the most recognized causes of skin barrier dysfunction and are considered as predisposing factors to AD [6]. Reportedly, tumor necrosis factor alpha (TNF-α), interleukin (IL)-4, and IL-13 are overexpressed in AD lesions and significantly down-regulate calcium-induced FLG expression in epidermal keratinocytes (KCs), which may contribute to skin barrier abnormalities [1, 13, 18]. Thus, skin barrier repair by blocking production of inflammatory cytokines that down-regulate FLG expression suggests a probable treatment strategy for AD.

Lidocaine is often clinically used as a short-acting local anesthetic and antiarrhythmic agent [40], as it possesses anti-inflammatory effects, and can be used as an immunomodulatory drug in treatment against allergic diseases [12, 29], as nebulized lidocaine was shown to be an effective and safe therapy in patients with mild-to-moderate asthma [14, 20, 39]. Recent studies have also demonstrated that lidocaine and its analogue JMF2-1 inhibited the activation of T lymphocytes and generation of important cytokines [17, 21, 30].

In the present study, we examined the effects of lidocaine on SEA- and SEB-stimulated cell proliferation and cytokine production in PBMCs from AD patients. Furthermore, the effect of lidocaine on FLG expression in HaCaT cells, a well-known immortalized human keratinocyte cell line, co-cultured with SE-activated PBMCs, was also examined.

## Materials and methods

### Materials

The following reagents were used: hydrochloride lidocaine (Sigma-Aldrich, St. Louis, MO, USA); SEA and SEB (Toxin Technology, Inc., Sarasota, FL, USA); cell lysis buffer for western blotting and immunoprecipitation (IP) analysis, phenylmethanesulfonyl fluoride (PMSF) and sodium dodecyl sulfate polyacrylamide gel electrophoresis (SDS-PAGE) sample loading buffer (5×) (Beyotime Institute of Biotechnology, Beijing, China); Dulbecco’s modified Eagle’s medium (DMEM)-high glucose, fetal calf serum (FCS), serum-free keratinocyte medium for culture of human KCs and Dispase II (GIBCO-BRL, Gland Island, NY, USA). If not otherwise stated, the reagents were obtained from Sigma-Aldrich Chemie GmbH (Deisenhofen, Germany).

### Subjects

Patient consent and ethical approval were obtained prior to the study. A total of ten AD patients (four males and six females; mean age, 27.75 years), who were admitted to our hospital, were included in the present study (Table 1). AD was diagnosed in accordance with the criteria of Hanifin and Rajka. The severity of disease was evaluated using the SCORing Atopic Dermatitis (SCORAD) index [34], which categorizes cases as mild (0–24 points), moderate (25–50 points) and severe (51–103 points). Venous blood samples were collected from AD patients and analyzed for total serum IgE level using a Pharmacia UniCAP-100 automatic immunoassay analyzer (Pharmacia Diagnostics AB, Uppsala, Sweden). None of the AD patients were currently administered with systemic steroids or immunosuppressant treatments, or utilizing potent topical steroids. This study was approved by the Ethics Committee of Shanghai Jiaotong University School of Medicine (Shanghai, China).

**Table 1:** Clinical data for the ten AD cases in the present study

Patient no.	Sex	Ethnicity	Age at onset (years)	SCORAD	Total serum IgE (IU/mL)
1	F	Han Chinese	29	15.1	110
2	M	Han Chinese	12	56.5	27,700
3	F	Han Chinese	67	27.5	830
4	F	Han Chinese	32	53	9,930
5	F	Han Chinese	10	38.5	2,160
6	M	Han Chinese	35	12.6	150
7	M	Han Chinese	9	49	8,710
8	F	Han Chinese	48	30.5	670
9	F	Han Chinese	25	54	16,100
10	M	Han Chinese	10	40.5	6,108

### PBMC purification

PBMCs were isolated from heparinized venous blood from AD patients on Ficoll-Hypaque gradients (Pharmacia, Uppsala, Sweden) and resuspended in Roswell Park Memorial Institute (RPMI) 1,640 medium supplemented with gentamicin (40 μg/mL) and 10 % pooled type AB normal human serum (Sigma-Aldrich). In all experiments, cells were cultured under the atmosphere containing 5 % CO_2_ at 37 °C.

### Analysis of lidocaine cytotoxicity on PBMCs

PBMCs (1 × 10^5^ cells/well) were cultured for up to 7 days in the presence or absence of lidocaine. Cell viability was determined using the Cell Counting Kit-8 (CCK-8) assay kit (Beyotime Institute of Biotechnology) according to the manufacturer’s instructions.

### Cellular proliferation and cytokine production analysis

Freshly isolated PBMCs (1 × 10^5^ cells/well) were cultured with SEA (100 ng/mL) and SEB (100 ng/mL) in the presence of different concentrations of lidocaine in 96-well plates for 7 days. Cells were pulsed with 1 μ Ci of tritiated methyl thymidine (Radiochemical Center, Amersham, UK) for the last 8 h of the culture period. The stimulation index was calculated by dividing the counts per million allergen-stimulated cultures by that of unstimulated cultures. For cytokine production analysis, PBMCs (1 × 10^6^ cells) were cultured with or without SEA (100 ng/mL) or SEB (100 ng/mL) for 72 h in the presence of different lidocaine concentrations. The levels of IL-4, IL-5, IL-13, IL-10, IL-12, IL-2, interferon gamma (IFN-γ), and TNF-α in the supernatant were measured with enzyme-linked immunosorbent assay (ELISA) kits (BioSource International, Inc., Camarillo, CA, USA).

### Co-culture experiment

HaCaT cells were grown in DMEM supplemented with 10 % FCS, 1 % penicillin–streptomycin, and differentiated in high CaCl_2_ (10 mM) to up-regulate FLG expression for 48 h at 37 °C and 5 % CO_2_, as previously described [13]. A co-culture system was established by culturing HaCaT cells and PBMCs using a polycarbonated trans-well insert membrane containing 0.4 μm pores (Becton-Dickinson & Company, Franklin Lakes, NJ, USA). Briefly, HaCaT cells (1 × 10^5^ cells/mL) were cultured at the bottom of 12-well plates, whereas PBMCs (2 × 10^6^ cells/mL) were cultured on the polycarbonated insert membranes. In the co-culture system, cells were cultured for 48 h in RPMI 1,640 medium supplemented with gentamicin (40 μg/mL) and 10 % pooled type AB normal human serum. The cells were then washed with phosphate-buffered saline and further incubated with serum-free medium for 24 h before stimulation. SEA and SEB were then added to the PBMCs alone or with lidocaine and cultured for an additional 72 h. As a control, HaCaT cells were cultured in 12-well plates with membrane inserts, but without PBMCs.

### RNA isolation and analysis

Total RNA was extracted from cells after treatment according to the experimental requirement. RNA extraction was performed using RNeasy Mini Kits (Qiagen, Valencia, CA, USA) according to the manufacturer’s guidelines. Total RNA (1 μg) was reverse-transcribed using the iScript cDNA Synthesis Kit (Bio-Rad, Hercules, CA, USA) at 25 °C for 5 min and 42 °C for 30 min, followed by 85 °C for 5 min in a final reaction volume of 40 μL. All assays were carried out under the following conditions: 35 cycles of denaturation at 95 °C for 15 s, followed by annealing and extension at 60 °C for 60 s using the ABI7300 Real Time PCR System (Applied Biosystems, Inc., Foster City, CA, USA). Melt curve analysis was performed to confirm the specificity of the amplified products. All samples were run in triplicate and relative expression was determined by normalizing samples to β-actin. Data were analyzed using the comparative ΔΔ*Ct* method. Primers and probes for human FLG and β-actin were purchased from Applied Biosystems.

### Western Blot analysis

For Western Blot analysis, protein extracts (30 μg) were prepared by lysing the cells in lysis buffer containing protease and phosphatase inhibitors, separated by SDS-PAGE, and transferred to polyvinylidene difluoride membranes. Membranes were blocked for 2 h in TBS [50 mM Tris–HCl (pH 7.5) and 150 mM NaCl] containing 0.1 % Tween 20 and 5 % non-fat dried milk. Mouse anti-human β-actin antibody (Santa Cruz Biotechnology, Santa Cruz, CA, USA) and mouse anti-human FLG antibody (Vector Laboratories, Inc., Burlingame, CA, USA) were used for western blotting. Subsequently, the membranes were incubated for 1 h with goat anti-mouse immunoglobulin (IgG) conjugated to horseradish peroxidase, rewashed, and developed using ECLTM reagents (Amersham Pharmacia Biotech, Inc., Piscataway, NJ, USA) and exposed to film.

### Statistical analysis

Results are presented as mean ± SEM. Significant differences between groups were examined using the Wilcoxon signed-rank test. A *p* value ≤0.05 was considered statistically significant.

## Results

### Cell viability

**Fig. 1:**
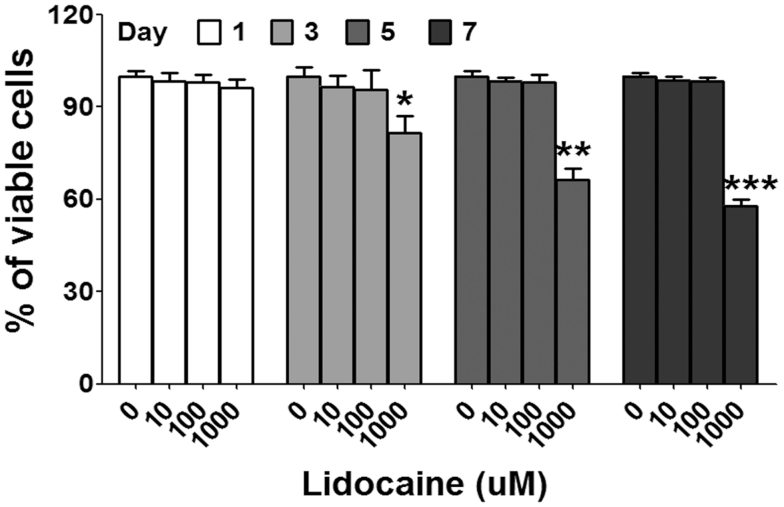
Changes in PBMC viability after incubation with lidocaine. PBMCs from ten AD patients were cultured for up to 7 days in the presence or absence of lidocaine. The percentage of viable cells decreased significantly following exposure to 1,000 μmol/L of lidocaine (*p < 0.05, **p < 0.01, and ***p < 0.001 vs. untreated control)

We initially investigated the cytotoxic effect of lidocaine on PBMCs from AD patients by culturing PBMCs in the presence or absence of different lidocaine concentrations for 7 days. Cell viability post-incubation was detected using the CCK-8 assay. We found that lidocaine at concentration up to 100 μmol/L did not affect PBMC viability, whereas concentrations at 1,000 μmol/L significantly decreased the percentage of viable cells (Fig. 1). Thus, subsequent experiments were performed with lidocaine concentrations ≤100 μmol/L.

### Lidocaine inhibited SEA- and SEB-induced T cell proliferation

**Fig. 2:**
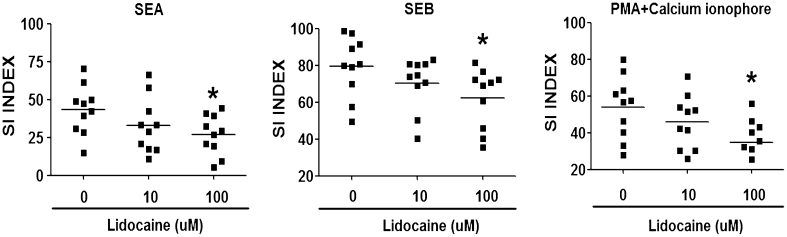
Proliferative responses of PBMCs from ten AD patients after stimulation with SEA, SEB, or PMA plus calcium ionophore in the presence or absence of lidocaine. The stimulation index (SI) was calculated. Proliferative responses were significantly decreased with 100 μmol/L of lidocaine. (Comparisons were between the two treated groups and un-treated control; *p < 0.05 and **p < 0.01, respectively)

PBMCs from ten AD patients were stimulated by SEA, SEB, or phorbol myristate acetate (PMA) plus calcium ionophores in the presence or absence of lidocaine. Cellular proliferation was assessed by tritiated thymidine incorporation and expressed as stimulation index (SI). Our results demonstrated that lidocaine at 100 μmol/L significantly inhibited the proliferative response of PBMCs stimulated by SEA or SEB (*p* < 0.05, Fig. 2). We also found that lidocaine at 100 μmol/L inhibited PMA/calcium ionophore-stimulated proliferative responses, which was in agreement with a previous report [39].

### Lidocaine suppressed cytokine production in SEA- and SEB-stimulated PBMCs

**Fig. 3:**
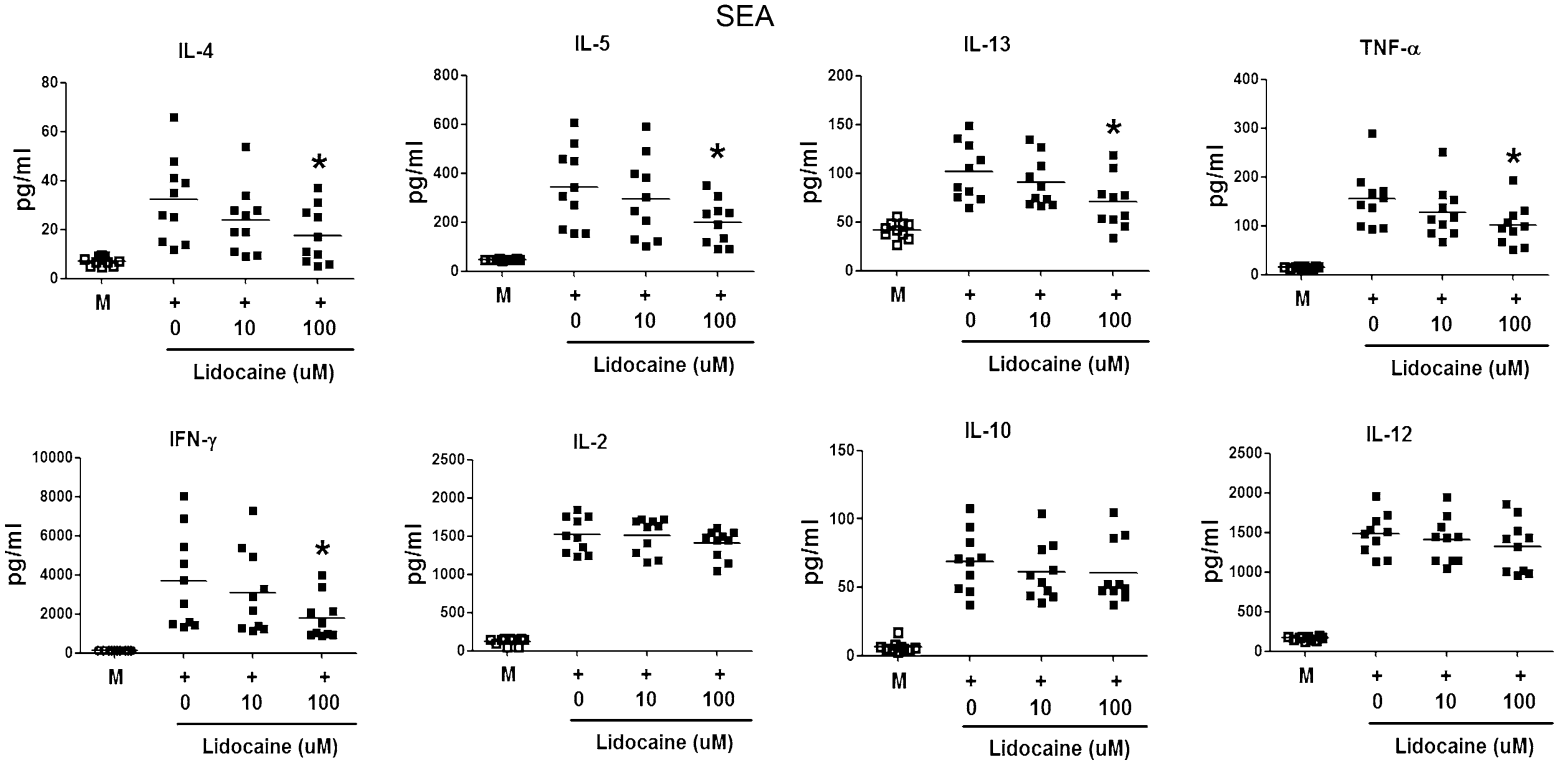
IL-4, IL-5, IL-13, TNF-α, IFN-γ, IL-2, IL-10, and IL-12 production by PBMCs from ten AD patients after SEA stimulation in the presence or absence of lidocaine measured via ELISA. Lidocaine (100 μmol/L) significantly decreased SEA-induced IL-4, IL-5, IL-13, TNF-α, and IFN-γ production in PBMCs from AD patients. (Comparisons were between the two treated groups and nontreated control, respectively, *p < 0.05, **p < 0.01, and ***p < 0.001 vs. untreated control)

**Fig. 4:**
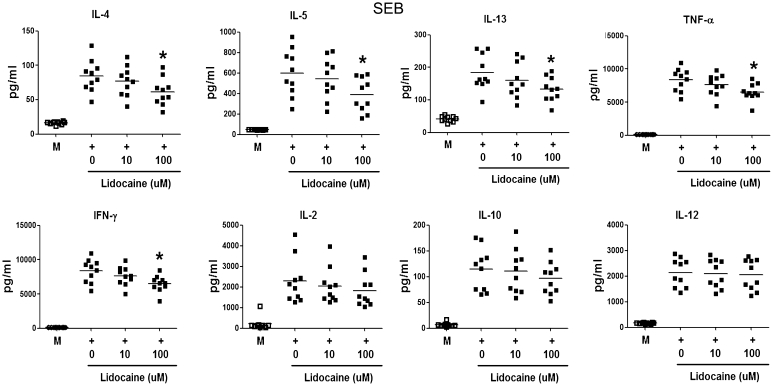
IL-4, IL-5, IL-13, TNF-α, IFN-γ, IL-2, IL-10, and IL-12 production by PBMCs from ten AD patients after stimulation with SEB in the presence or absence of lidocaine measured via ELISA. Lidocaine (100 μmol/L) significantly decreased SEB-induced IL-4, IL-5, IL-13, TNF-α, and IFN-γ production in PBMCs from AD patients. (Comparisons were between the two treated groups and nontreated control, respectively, *p < 0.05, **p < 0.01, and ***p < 0.001)

Next, we tested the ability of lidocaine to reduce cytokine production in PBMCs from AD patients stimulated by SEA or SEB. Stimulation with SEA or SEB increased release of IL-4, IL-5, IL-13, TNF-α, IFN-γ, IL-2, IL-10, and IL-12 in PBMCs from AD patients, whereas lidocaine (100 μmol/L) significantly reduced IL-4, IL-5, IL-13, TNF-α, and IFN-γ production following SEA or SEB stimulation (*p* < 0.05, Figs. 3, 4). However, no similar inhibitory effects of lidocaine on IL-2, IL-10, and IL-12 production were observed (Figs. 3, 4).

### Lidocaine blocked the down-regulation of FLG expression in HaCaT cells co-cultured with SEA- and SEB-activated PBMCs

**Fig. 5:**
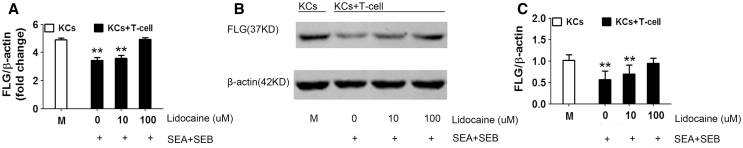
The effects of lidocaine on FLG expression in HaCaT cells were studied in a trans-well co-culture system. PBMCs from ten AD patients were seeded on the surface of insert membrane and HaCaT cells were seeded on the bottom of the plate. FLG expression was analyzed using real-time RT-PCR and western blotting. Lidocaine significantly inhibited FLG down-regulation in HaCaT cells co-cultured with PBMCs in the presence of SEA and SEB at both the mRNA (**a**) and proteins (**b**) levels. **c** Densitometric analysis of the western blotting results is shown, in which the intensity of the protein signal normalized against the β-actin signal. (Comparisons were between the two treated groups and nontreated control, respectively, *p < 0.05 and **p < 0.01)

Next, we further investigated the effect of lidocaine on FLG expression in HaCaT cells co-cultured with SEA- and SEB-activated PBMCs and found that FLG mRNA levels in HaCaT cells co-cultured with activated PBMCs were significantly lower than those in HaCaT cells cultured alone. In the co-culture system, the addition of lidocaine (100 μmol/L) to PBMCs blocked the down-regulation of FLG expression, both at the mRNA (Fig. 5a) and protein levels (Fig. 5b, c), following induction by SEA- and SEB-activated PBMCs.

## Discussion

To the best of our knowledge, the present study is the first to demonstrate lidocaine inhibition of PBMC proliferation in response to SEA and SEB stimulation. In addition, the production of both T_H_1-type cytokines (TNF-α and IFN-γ) and T_H_2-type cytokines (IL-4, IL-5, and IL-13) from SEA and SEB-activated PBMCs was inhibited by lidocaine at a concentration of 100 μmol/L. These new findings indicate that lidocaine may exert immunoregulatory effects on immune cells and can be used as an anti-inflammatory agent in the treatment of AD.

The skin lesions present in AD are frequently colonized with *S. aureus* strains that produce SsAgs [23, 43] and recent evidence has indicated that one type of skin-homing T cell (CD4 +/Foxp3 +) in AD patients exerts effector T_H_2-like functions promoting SsAg stimulation, which may aggravate allergic skin inflammation [25]. SsAgs also have the ability to augment antigen-specific T_H_1 responses by stimulating antigen-presenting cells, which might contribute to AD chronification [2], and can contribute to AD pathogenesis by increasing the frequency of memory T cells that are able to migrate to and be activated within AD lesions, where they directly contact epidermal KCs [38]. Several cytokines, including TNF-α, IL-4, and IL-13, are released from T cells and monocytes and were found to down-regulate FLG expression in KCs [13, 18], thus contributing to compromised skin barrier function.

*Staphylococcus aureus* can colonize AD skin and simultaneously secrete SEA and SEB, which can subsequently induce the release of TNF-α, IL-4, and IL-13 from immune cells that, in turn, can down-regulate FLG expression in KCs. Thus, we evaluated the inhibitory effect of lidocaine on activated PBMCs via measuring FLG expression in HaCaTs and PBMCs in a co-culture system in which PBMCs were stimulated with SEA and SEB together to mimic an in vivo environment. Our results showed that lidocaine significantly blocked FLG down-regulation in HaCaT cells co-cultured with SEA and SEB-stimulated PBMCs. We ascribe this effect of lidocaine to its inhibition of cytokine production (i.e., TNF-α, IL-4, and IL-13) in activated PBMCs, suggesting that lidocaine may exert an inhibitory effect on inflammatory T cells activated by SE, which, in turn, provide beneficial effects to skin barrier repair.

Glucocorticosteroids are commonly used drugs for AD treatment, although corticosteroid treatment has been challenged for its adverse effects on the skin barrier. Topical corticosteroid application can delay epidermal barrier restoration, lead to epidermal thinning, and even induce a strong reduction in human beta-defensin production [16, 31, 36]. In this context, lidocaine, with its inhibitory effect on PBMCs and potential to improve skin barrier function, may be used as a novel treatment against AD. Nevertheless, the use of lidocaine–prilocaine cream (a eutectic mixture of local anesthetics) in pediatric and dermatologic practice to obtain local anesthesia, can also cause many skin side effects, which include transient skin blanching, erythema, urticaria, allergic contact dermatitis, irritant, contact dermatitis, hyperpigmentation, and purpura [4, 7, 10, 28, 33]. Thus, further studies on animals or patients are still needed and the safety of lidocaine for the treatment of AD should be evaluated as well.

Previous studies have demonstrated that lidocaine can relax smooth airway muscles by decreasing intracellular Ca^2+^ concentrations [15]. Activation of Ca^2+^, K^+^, and Cl^−^ channels is important for activation of T-cells in the early phase of an immune reaction [35, 41]. Elevation of Ca^2+^ plays a critical role in the activation and translocation of nuclear transcription factors, including nuclear factor (NF) AT, NF-κB, and c-Jun N-terminal kinase (also known as mitogen-activated protein kinase 8) [5, 11]. Moreover, recent studies have reported that lidocaine attenuates lipopolysaccharide-induced acute lung injury and inhibits epithelial chemokine secretion through inhibition of NF-κB activation [9, 22], thus, inhibition of cytokine production and T cell proliferation by lidocaine is likely related to its down-regulation of NF-κB signaling [21]. Therefore, the inhibitory effects of lidocaine on SEA- and SEB-induced PBMC proliferation and cytokine production may occur through the inhibition of the NF-κB pathway; however, the precise underlying molecular mechanism(s) require further exploration.

In conclusion, herein, we demonstrated the inhibitory effects of lidocaine against SEA- and SEB-stimulated activation of PBMCs from AD patients. Therefore, lidocaine is a promising anti-inflammatory agent in the treatment of AD.
